# Exploring the Synergistic Secretome: Insights from Co-Cultivation of *Aspergillus brasiliensis* and *Trichoderma reesei* RUT-C30

**DOI:** 10.3390/jof10100677

**Published:** 2024-09-28

**Authors:** Guilherme Bento Sperandio, Reynaldo Magalhães Melo, Taísa Godoy Gomes, Robert Neil Gerard Miller, Luis Henrique Ferreira do Vale, Marcelo Valle de Sousa, Carlos André Ornelas Ricart, Edivaldo Ximenes Ferreira Filho

**Affiliations:** 1 Laboratory of Enzymology, Department of Cell Biology, University of Brasília, Brasília 70910-900, DF, Brazil; guibsp093@gmail.com (G.B.S.); eximenes@unb.br (E.X.F.F.); 2 Laboratory of Protein Chemistry and Biochemistry, Department of Cellular Biology, University of Brasília, Brasilia 70910-900, DF, Brazil; reynaldommelo@gmail.com (R.M.M.); luisvale@unb.br (L.H.F.d.V.); mvsousa@unb.br (M.V.d.S.); 3 Laboratory of Microbiology, Department of Cell Biology, University of Brasília, Brasilia 70910-900, DF, Brazil; taisa.godoy@gmail.com (T.G.G.); robertmiller@unb.br (R.N.G.M.)

**Keywords:** secretome, co-culture, fungal enzymes, lignocellulosic biorefineries, proteomics, sugarcane bagasse

## Abstract

The spectrum of enzymes required for complete lignocellulosic waste hydrolysis is too diverse to be secreted by a single organism. An alternative is to employ fungal co-cultures to obtain more diverse and complete enzymatic cocktails without the need to mix enzymes during downstream processing. This study evaluated the co-cultivation of *Aspergillus brasiliensis* and *Trichoderma reesei* RUT-C30 in different conditions using sugarcane bagasse as the carbon source. The resulting enzymatic cocktails were characterized according to the impact of strain inoculation time on enzymatic activities and proteome composition. Data revealed that the profile of each enzymatic extract was highly dependent on the order in which the participating fungi were inoculated. Some of the co-cultures exhibited higher enzyme activities compared to their respective monocultures for enzymes such as CMCase, pectinase, β-glucosidase, and β-xylosidase. Analysis of the *T. reesei* RUT-C30 and *A. brasiliensis* co-culture secretome resulted in the identification of 167 proteins, with 78 from *T. reesei* and 89 from *A. brasiliensis*. In agreement with the enzymatic results, proteome analysis also revealed that the timing of inoculation greatly influences the overall secretome, with a predominance of *T. reesei* RUT-C30 proteins when first inoculated or in simultaneous inoculation.

## 1. Introduction

Lignocellulosic biorefineries are industrial plants designed to produce various consumer goods, such as biofuels and basic chemicals, using low-value-added agro-industrial waste as raw material [1]. Biorefineries represent a result of significant scientific effort to promote a sustainable bioeconomy and reduce dependency on non-renewable, highly polluting fossil fuels. To make subsequent processes viable, agroindustrial residues composed of lignocellulose must first undergo hydrolysis to transform their constituent polysaccharides into fermentable sugars. However, one of the greatest bottlenecks in this industry is the high cost of the enzymes required for hydrolysis [2].

Hydrolytic enzymes employed in biorefineries usually come from microbial sources, with filamentous fungi being the main industrial producers. The most widely utilized industrial strain to date for commercial hydrolytic enzyme production is the hypersecreting mutant filamentous fungus *Trichoderma reesei* (*Hypocrea jecorina*) RUT-C30. The spectrum of enzymes required for complete lignocellulosic waste hydrolysis is, however, too diverse to be secreted by a single organism alone. Even the high-cellulase-producing strain *T. reesei* RUT-C30 does not secrete a complete enzymatic cocktail, with only a low production of β-glucosidases in comparison with its ability to produce other hydrolases [3]. To circumvent such limitations, it is possible to employ fungal co-cultures to obtain improved enzymatic cocktails without the need to mix enzymes during downstream processing. In such a system, two or more fungi are grown simultaneously, sharing the same fermentation apparatus. The cooperation or antagonism occurring between these fungi is then expected to modify certain enzymatic activities relative to their monocultures. Several studies on fungal co-cultivation have already been published and compiled in a previous publication [4]. However, the application of co-cultures for lignocellulolytic enzyme production is still a largely unexplored area in industrial microbiology. *Aspergillus brasiliensis* (Phylum Ascomycota, Order Eurotiales) is a cosmopolitan fungus [5] originally isolated from various Brazilian soils but also found in soils from Australia, the USA, and the Netherlands. *A. brasiliensis* belongs to the section Nigri, which includes *Aspergillus* species with black spores. As the member species display similar morphological characteristics, taxonomic identification is a considerable challenge. The Nigri section currently includes members of significant industrial, medical, and ecological interest and comprises over 25 species, among which notable members include *A. niger*, *A. fumigatus* and *A. nidulans* [6].

The decomposition of recalcitrant dead plant matter is typically carried out in natural environments by a diverse community of microorganisms, of which filamentous fungi are the primary players [7]. However, in industrial processes for the production of lignocellulolytic enzymes, monoculture is the standard technique, as it is well-established and simple to control. As a consequence, current cocktails need to be enriched with enzymes from additional fungi to achieve satisfactory levels of hydrolysis. This culminates in an expensive downstream process that considerably raises production costs.

The proteins secreted by a cell, tissue or organism comprise a subproteome, which is referred to as a secretome. A review on secretome analysis in several fungi belonging to the *Trichoderma* and *Aspergillus* genera under various monoculture conditions has been published previously [8]. In the present article, we performed a characterization of fungal co-cultures through proteomic and enzymatic approaches, examining the strains *T. reesei* RUT-C30 and *A. brasiliensis* under various growth conditions.

## 2. Materials and Methods

### 2.1. Origin and Maintenance of Fungi

*T. reesei* RUT-C30 was kindly provided by Dr. Roberto do Nascimento Silva from the Faculty of Medicine at the University of São Paulo (Ribeirão Preto, SP, Brazil). *A. brasiliensis* was originally isolated from the soil of the Brazilian Savanna (Cerrado) and deposited in the fungal culture collection center at the Enzymology Laboratory, University of Brasilia, Brazil (genetic heritage number: 010237/2015-1). This strain was also stored in the Culture Collection for Microorganisms for Control of Plant Pathogens and Weeds, maintained at the Brazilian Agricultural Research Corporation (Embrapa, Brazil). This collection is registered in the CCINFO database (MCPPW 1128) (URL: https://ccinfo.wdcm.org/details?regnum=1128 accessed on 20 July 2024). Fungal stocks were maintained at −80 °C in 50% (*v*/*v*) glycerol. *A. brasiliensis* and *T. reesei* RUT-C30 were identified at the molecular level through specific PCR amplification and sequence analysis of ribosomal DNA internal transcribed spacer regions (rDNA ITS) and partial gene regions encoding the translation elongation factor (TEF1α), RNA polymerase II (RPB2), calmodulin (CAL) and actin (ACT) as appropriate DNA barcoding markers to confirm fungal identity. For identification, consensus sequences were compared with non-redundant sequences in the NCBI nucleotide database using the BLAST algorithm [9]. All DNA barcoding markers confirmed species identity, with representative sequences deposited in NCBI under accession numbers OR542772 (*A. brasiliensis*—RPB2 gene), OR467038 (*A. brasiliensis*—rDNA ITS), OR542773 (*T. reesei* RUT-C30—RPB2 gene) and OR467037 (*T. reesei* RUT-C30—rDNA ITS).

### 2.2. Fungal Cultivation

All reagentes were purchased from (Sigma-Aldrich St. Louis, MO, USA), a subsidiary company of Merck KGaA, unless otherwise specified. Sugarcane bagasse (SCB) was acquired from a local supplier in the state of Goiás, Brazil. SCB was employed as the sole carbon source throughout all experiments. To allow a simulation of degradation of the lignocellulosic material as it would naturally occur if discarded in the environment, SCB was not pre-treated. A volume of 100 mL of the supplemental medium (7 g/L KH_2_PO_4_; 2 g/L K_2_HPO_4_; 0.5 g/L MgSO_4_ 7H_2_O; 1 g/L (NH_4_)_2_SO_4_; 0.6 g/L Yeast Extract, and 1% (*w*/*v*) SCB) was added to 250 mL Erlenmeyer flasks, which were then autoclaved at 121 °C for 20 min. *T. reesei* RUT-C30 and *A. brasiliensis* were grown on Petri dishes containing 2% malt extract agar until sporulation, which ranged from 7 to 12 days. Spores were manually scraped off culture plates using a glass microscopy slide and then immersed in 50 mL of 0.9% (*w*/*v*) NaCl. Following spore counting using a Neubauer chamber, specific inoculation volumes were prepared to result in a final concentration of 10^5^ spores/mL in flasks containing the supplemental medium. For monocultures, inoculum originated from a single fungus (*T. reesei* RUT-C30 or *A. brasiliensis*). In the case of co-cultures, 50% of the inoculum concentration originated from each participating fungus (i.e., 5 × 10^4^ spores/mL for each fungus), thus maintaining a final total concentration of 10^5^ spores/mL in the medium under both tested conditions.

The pH of the *T. reesei* RUT-C30 cultivation in supplemental medium was monitored daily during 9 days with no significant variation in the pH of system observed, remaining constant at pH 6.0 during the entire cultivation period.

### 2.3. Enzyme Production

Liquid cultures inoculated with spores were placed on automatic shakers and incubated at 120 rpm and 28 °C for nine days. Cultures were performed in biological triplicate. Every 48 h, 1 mL aliquots of spent culture media were collected from each Erlenmeyer flask for the construction of enzymatic induction curves. Following collection, all aliquots were frozen at −20 °C for subsequent analysis. After the nine-day incubation period, spent cultures were filtered using cellulose-free synthetic tissue. The filtrate containing enzymatic activities was collected and referred to as Crude Extract (CE). CE was frozen and stored at −20 °C for further enzymatic assays and proteome analysis, as described below. In addition to analysis of individual monocultures of *T. reesei* RUT-C30 and *A. brasiliensis*, and co-cultures of both fungi inoculated simultaneously, other configurations were also tested, as presented in Table 1.

### 2.4. Enzyme Characterization

Xylanase, mannanase, carboxymethylcellulase (CMCase), pectinase, β-xylosidase, β-glucosidase, β-mannosidase, and β-galactosidase activities were determined as described elsewhere [10,11]. The substrates used were oat spelt xylan, mannan, carboxymethyl cellulose, pectin, p-nitrophenyl-β-D-xylopyranoside, p-nitrophenyl-β-D-glucopyranoside, p-nitrophenyl-β-D-mannopyranoside and p-nitrophenyl-β-D-galactopyranoside, respectively. Enzyme activities (IU/mL) were expressed as µmol of product formed per min per mL of enzyme solution. Specific activities were expressed in IU/mg of total protein. Protein concentration was determined using the Bradford assay [12], with bovine serum albumin employed as standard. The effect of temperature on enzymatic activity was assessed by conducting enzyme assays as described above, with a variation in temperature during the incubation period. Temperatures of 30, 40, 50, 60, 70, and 80 °C were tested. Evaluation of temperature effects on enzymatic activity was performed using the CE on the 9th day of cultivation. A modified protocol of Miller’s DNS method was employed to evaluate the influence of pH (range 3–10) on CMCase activity. The reaction mix consisted of 5 µL of substrate at a concentration of 2%, 5 µL of enzyme (CE), and 5 µL of buffer. The buffers employed were as follows: sodium citrate (pH values 3, 4 and 5), sodium phosphate (pH values 6, 7 and 8), and glycine (pH values 9 and 10), all at a concentration of 50 mM. The effect of pH on CMCase activity was evaluated using CE on the 9th day of cultivation. Saccharification assays were conducted to test the ability of crude extracts to hydrolyze lignocellulosic biomass. Assays were performed on a microscale, utilizing 1.5 mL tubes containing 0.01 g of sugarcane bagasse (1% *m*/*v*) and 20 µg of protein from each culture. The volume was adjusted to 1 mL with a 100 mM pH 5.0 sodium acetate buffer. Samples were incubated at 50 °C with stirring at 720 RPM in a Vortemp incubation shaker (Labnet). Tests were conducted after 4, 8, 12, and 24 h of incubation. The reducing sugars released by the enzymes were measured using the DNS reagent method [11], with results compared to a standard glucose curve.

### 2.5. Sample Preparation for Mass Spectrometry

CE samples (1 mL) were precipitated using four volumes of ice-cold acetone (−20 °C) and incubated overnight [13]. Pellets were solubilized in 0.1 M Tris, 6 M guanidine-HCl, pH 7.6 and protein concentration quantified using the Pierce BCA assay kit (Thermo Scientific, Bremen, Germany). To reduce protein disulfide bridges, 40 µg of sample was mixed with 5 mM DL-dithiothreitol (DTT) and incubated at 55 °C for 25 min. Samples were then alkylated with 14 mM iodoacetamide in the dark for 40 min. The alkylation reaction was quenched by adding DTT to a final concentration of 10 mM. Proteins were diluted six-fold with a 0.1 M ammonium bicarbonate buffer, at pH 7.9, then submitted to proteolysis at a 1:50 trypsin/protein ratio at 37 °C for 16 h. To stop the proteolysis, 0.5% (*v*/*v*) trifluoroacetic acid (TFA) was added to the samples. Peptides were desalinated using a 200 µL low-binding tip containing a slurry of oligo-R2 and oligo-R3 resins in a 1:1 ratio and an Empore C18 disk (CDS Analytical LLC, Oxford, MS, USA). Desalting columns were first conditioned with 150 µL of methanol, followed by washing with 200 µL of 0.1% TFA in acetonitrile (ACN) and 200 µL of 0.1% TFA in water. Peptide samples were then loaded onto the columns and washed with 200 µL of 0.1% TFA in water. To elute the peptides, the columns were sequentially washed with 40 µL of 0.1% TFA in 20% ACN, 40 µL of 0.1% TFA in 50% ACN and 90 µL of 0.1% TFA in 90% ACN. The eluted peptides were dried using a vacuum concentrator and resuspended in 15 µL of injection solution containing 2% ACN and 0.1% formic acid. Finally, the prepared peptide samples were subjected to liquid chromatography coupled to mass spectrometry (LC-MS/MS) for analysis.

### 2.6. LC-MS/MS

LC-MS/MS analysis was carried out on a nano-UHPLC Dionex Ultimate 3000 system coupled to an Orbitrap Elite™ mass spectrometer (Thermo Scientific, Bremen, Germany). Chromatographic separation utilized an analytical column (75 μm × 30 cm) packed with Reprosil-Pur 120 Å pore C18 particles of 3 µm (Dr. Maisch, Ammerbuch, Germany), together with a trap column (100 μm × 3 cm) packed with Reprosil-Pur 120 Å pore C18 particles of 3 µm (Dr. Maisch). Elution of peptides was achieved by employing two LC solvents: solvent A (0.1% formic acid) and solvent B (0.1% formic acid in ACN). The flow rate was set at 0.250 μL/min, with a total runtime of 116 min. The following gradient was employed: 0–10 min 5% solvent B; 10–17.5 min: 9% solvent B; 17.5–65 min: 25% solvent B; 65–90 min: 45% solvent B; 90–91 min: 85% solvent B; 91–96 min: 85% solvent B; 91–96 min: 2% solvent B; 96–116 min: 2% solvent B. The acquisition of MS data was performed for 106 min. A data-dependent acquisition strategy was employed, with a dynamic exclusion of 90 s and an isolation window of 2 *m*/*z*. Higher energy collision dissociation (HCD) was employed for peptide fragmentation with a normalized energy of 35% applied to the 15 most abundant peaks of each MS1 scan. The resolutions utilized were 120,000 FWHM (Full Width at Half Maximum) for MS1 and 15,000 FWHM for MS2.

### 2.7. Bioinformatics and Statistical Analysis

Identification and quantification of proteins were performed using MetaMorpheus software (v1.0.2) [14]. Raw files were loaded into MetaMorpheus along with reference proteomes from *T. reesei* RUT C-30 (strain ATCC 56765/BCRC 32924/NRRL 11460/RUT C-30) and *A. brasiliensis* (strain CBS 101740/IMI 381727/IBT 21946) obtained from UniProt (April 2023), to enable construction of a concatenated database along with common contaminants. Data processing began with spectrum calibration using default parameters. This was followed by Global-enhanced Post-translational Modification Discovery (G-PTM-D), which searched for common biological PTMs, N-glycosylation, O-glycosylation and other types of glycosylation. In the final step, peptides and proteins were searched and quantified using the calibrated spectra and a database incorporating the PTMs discovered by G-PTM-D. Quantification was performed using FlashLFQ, an internal tool within MetaMorpheus (v1.0.2) [15], which employs enhanced match between runs. The interpretation of identifications and statistical protein quantification analysis was conducted in RStudio (v2023.12.1) (RStudio Team, 2021), utilizing the R programming language (v4.3.3) (R Core Team, 2022). Specifically, the pmartR package (v2.4.5) was employed for quality control and statistical proteomics data analysis [16]. The data were analyzed using one-way ANOVA, followed by post hoc Tukey’s tests, to assess the differences between samples. A level of *p* < 0.05 was employed for all statistical analyses.

## 3. Results and Discussion

### 3.1. Enzyme Activity Profiles

The identities of *T. reesei* RUT C-30 and *A. brasiliensis* were confirmed at the molecular level, with representative sequences deposited in NCBI as described in Section 2. In addition to investigating enzymatic induction during co-culture over time, this study also examined monocultures and the impact of different colonization time periods between both fungi in co-cultivation, as summarized in Table 1.

**Figure 1:**
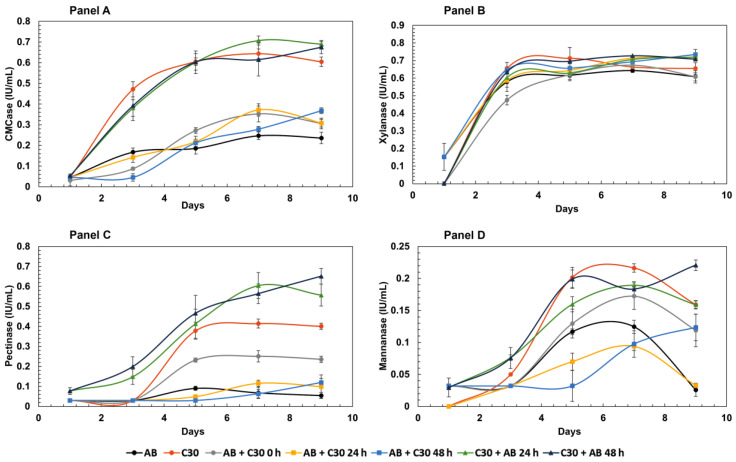
Enzymatic activity profiles tested on natural substrates obtained over 9 days in mono- and co-cultures of *A. brasiliensis* and *T. reesei* RUT-C30 grown in liquid medium with sugarcane bagasse as sole carbon source, as described in Table 1. The substrates used in the enzymatic assays were Panel (**A**) = carboxymethyl cellulose; Panel (**B**) = oat spelt xylan; Panel (**C**) = pectin and Panel (**D**) = mannan. Error bars represent the standard deviation between three biological replicates. All experiments showed *p* < 0.05 (ANOVA).

Figure 1 displays enzymatic activities using xylan, mannan, carboxymethylcellulose (CMC) and pectin. The highest CMCase activity was observed for co-cultures of *T. reesei* RUT-C30 where *A. brasiliensis* inoculation was staggered for 24 h and 48 h (C30 + AB 24 h and C30 + AB 48 h, respectively). Considering the standard deviations, it can be concluded that both co-cultures produced similar results. Performances for both co-culture models were approximately 10% higher than observed for the *T. reesei* RUT-C30 monoculture at the final nine-day time point. One possible explanation for such findings is that β-glucosidase from *A. brasiliensis* may supplement the typical low production of this enzyme by *T. reesei* RUT-C30. Such a supplementation might prevent events such as CMCase inhibition by its hydrolysis products, thereby enhancing the release of reducing sugars.

Previous studies have shown an increase in cellulase activities in co-cultures of *T. reesei* RUT-C30 with other Aspergillus fungi, including *A. niger*, under different cultivation conditions [17]. Zhao et al. [18] also demonstrated a 40% increase in CMCase activity in co-cultures of *T. reesei* RUT-C30 and *A. niger* compared to the monoculture of *T. reesei*. Regarding xylanase activity, minimal differences were observed among the tested cultures, and from the fourth day onwards, activities of all cultures became indistinguishable. On the other hand, pectinase activity of co-cultures of *T. reesei* RUT-C30 inoculated with *A. brasiliensis* after 24 h and 48 h exhibited a notable performance, similar to that observed for CMCase activity. These co-cultures displayed pectinase activities that were 37% and 48% higher than the *T. reesei* RUT-C30 monoculture, respectively. Considering the nearly absent production of pectinases by the monoculture of *A. brasiliensis*, these co-cultures demonstrated a 700% increase in this activity when compared to the *A. brasiliensis* monoculture. Such activities described here are relevant, given that pectinases are enzymes with considerable importance for the fruit pulp and juice industry [19], as well as with applications in tea, coffee and even textile industries [20]. Mannanase activity showed the lowest overall performance among the mono- and co-cultures. Co-culture of *T. reesei* RUT-C30 inoculated after 48 h with *A. brasiliensis* exhibited noteworthy results, however, with values close to 0.25 IU/mL. The lower values for pectinase and mannanase activities can be attributed to the composition of sugarcane bagasse, which contains smaller amounts of their respective substrates within the sugarcane bagasse cell wall.

**Figure 2:**
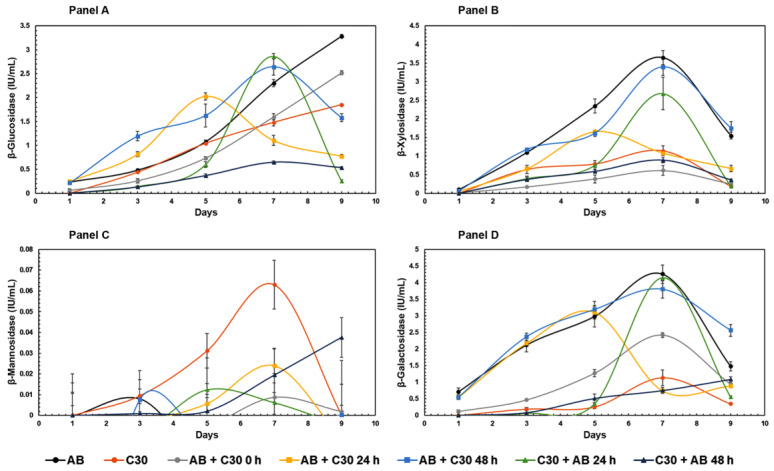
Enzymatic activity profiles tested on synthetic substrates, obtained over 9 days in mono- and co-cultures of *A. brasiliensis* and *T. reesei* RUT-C30 grown in liquid medium with sugarcane bagasse as sole carbon source, as described in Table 1. The substrates used in the enzymatic assays were: Panel (**A**) = p-nitrophenyl-β-D-glucopyranoside, Panel (**B**) = p-nitrophenyl-β-D-xylopyranoside, Panel (**C**) = p-nitrophenyl-β-D-mannopyranoside, Panel (**D**) = p-nitrophenyl-β-D-galactopyranoside. Error bars represent the standard deviation between three biological replicates. All experiments showed *p* < 0.05 (ANOVA).

When evaluating activities tested on synthetic substrates, the co-cultures demonstrated significant β-glucosidase, β-xylosidase, and β-galactosidase activities (Figure 2). As expected, the monoculture of *T. reesei* RUT-C30 displayed the lowest β-glucosidase activity, a well-known characteristic for this strain that is widely reported in the literature [3,21]. Conversely, the monoculture of *A. brasiliensis* exhibited a 57% higher β-glucosidase activity than *T. reesei* RUT-C30 on the final day of analysis. *A. brasiliensis*, given its ability to produce this enzyme, offers potential as a non-mycotoxin-producing alternative to the commonly employed *A. niger*, which is often partnered with *T. reesei* RUT-C30 to enhance β-glucosidase activity in co-cultivation cocktails [18,22]. The co-culture format that exhibited the greatest potential for increasing β-glucosidase activity was achieved through simultaneous inoculation of the two fungi (AB + C30 0 h), resulting in an intermediate level between the activities of *A. brasiliensis* and *T. reesei* RUT-C30, with a 38% increase compared to the latter fungus in monoculture. When comparing inoculation delay experiments on β-glucosidase activity, where *T. reesei* RUT-C30 was the first colonizer, a negative impact on enzyme activity was observed. Experiments with *A. brasiliensis* inoculated with delays of 24 h and 48 h compared to *T. reesei* RUT-C30 showed decreases in β-glucosidase activity on the final day of cultivation. For the co-culture where *T. reesei* RUT-C30 was employed as the first colonizer, followed by *A. brasiliensis* after 24 h (C30 + AB 24 h), a significant increase in enzymatic activity was observed between days 5 and 7, although this value dropped substantially on the final day of incubation.

*A. brasiliensis* monoculture resulted in the highest β-xylosidase activity, consistent with previous studies that have demonstrated its ability to produce this enzyme when grown on lignocellulosic substrates [23]. Co-cultures of *A. brasiliensis*, with *T. reesei* RUT-C30 inoculated after 48 h, resulted in β-xylosidase activity values similar to those of the monoculture of *A. brasiliensis*. The co-culture of simultaneous inoculation (AB + C30 0 h) performed similarly to that of the monoculture of *T. reesei* RUT-C30, secreting approximately 0.25 IU/mL. Co-culture with a 48 h delay resulted in β-xylosidase activities 696% higher than observed in simultaneous cultivation and monoculture of *T. reesei* RUT C-30. One possible explanation for this increase in activity may reflect the close evolutionary relationship between *A. brasiliensis* and *A. niger*. The latter species is known to acidify growth media to create preferred growing conditions. This is particularly common in the absence of competition and during the initial stages of cultivation, with reports of acidification also influencing enzymatic activities [24]. *A. brasiliensis* may potentially share a similar capacity. These results also highlight the impact of the time intervals between inoculations. Cultures of *A. brasiliensis* subsequently inoculated with *T. reesei* RUT-C30 after a shorter interval (24 h) exhibited a completely different β-xylosidase induction curve, with a sharp drop in activity on the ninth day of cultivation. These data suggest influencing effects on the first day when *T. reesei* RUT-C30 is present, decreasing β-xylosidase activity. The negative effect of *T. reesei* RUT-C30 on β-xylosidase activity is further confirmed by the other experimental setup where *T. reesei* RUT-C30 is the initial colonizer, with *A. brasiliensis* subsequently inoculated. In all cultures where *T. reesei* RUT-C30 is cultivated for a longer period than *A. brasiliensis*, β-xylosidase activities were lower than when the opposite scenario was employed. Unfortunately, the available literature on β-xylosidase activities in fungal co-cultures is limited. One of the few investigations of β-xylosidases in co-cultures is the study by Hu et al. [25], where the co-culture between *A. niger* and *A. oryzae* resulted in a 55% decrease in β-xylosidase activity when compared to the monoculture of *A. niger*. The scarcity of information regarding this enzymatic activity in co-cultures not only hinders direct comparisons but also underscores the significance of the present study for co-cultures.

Regarding *β-galactosidase*, the AB + C30 48 h co-culture showed the highest activity. Similar to β-glucosidase activities and, to a lesser extent, β-xylosidase, there was also a peak in activity on the seventh day of cultivation. Due to logistical reasons, all the characterizations mentioned in the following sections of this study were conducted using samples from the final day, even though it may not always be the day with the highest activity, as observed for β-glucosidase. Finally, no significant activity for β-mannosidases was detected, similar to the findings in the monoculture experiments for *A. brasiliensis*.

### 3.2. Effect of Temperature on the Enzymatic Activity

Crude extract derived from co-cultures is a complex mixture that represents the contributions from each of the different fungi present. Currently, there is limited information in the literature regarding how physical or chemical conditions can affect the enzymatic activity of co-cultures.

**Figure 3:**
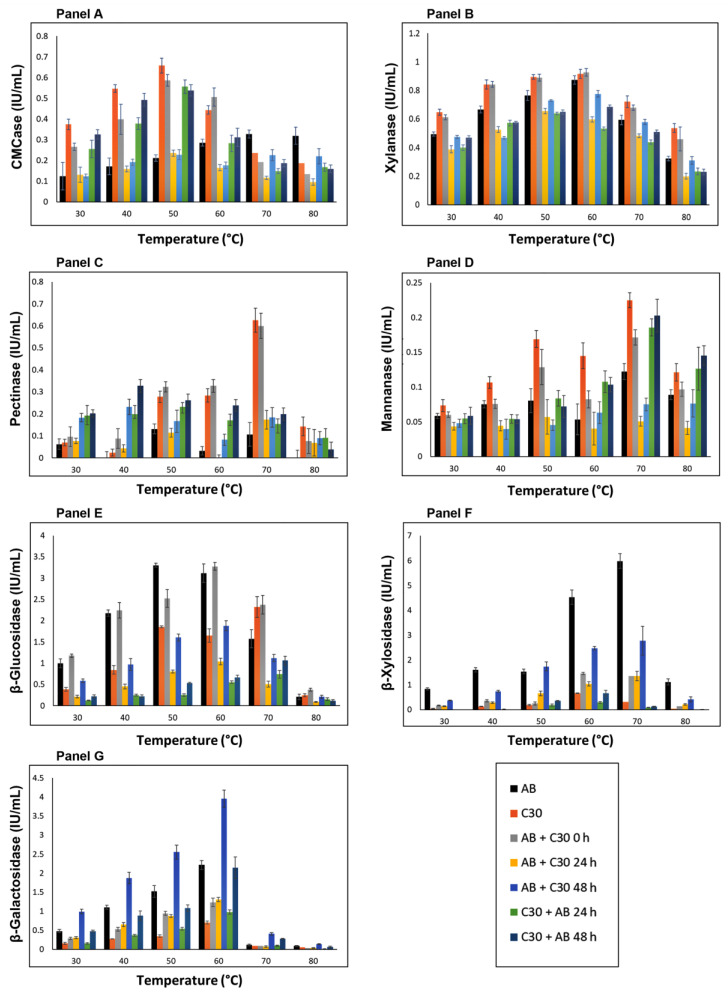
Influence of temperature on the enzymatic activity of crude extracts from monocultures of *A. brasiliensis* and *T. reesei* RUT-C30 and their simultaneous co-cultures and with different inoculation times, as described in Table 1. Panel (**A**) = CMCase activity, Panel (**B**) = Xylanase activity, Panel (**C**) = Pectinase activity, Panel (**D**) = Mannanase activity, Panel (**E**) = β-glucosidase activity, Panel (**F**) = β-Xylosidase activity and Panel (**G**) = β-galactosidase activity. The error bars represent the standard deviation between three biological replicates. All experiments showed *p* < 0.05 (ANOVA).

The catalytic activity of enzymes can be affected by the environmental temperature. When considering crude fungal extracts, enzyme activities are measured for not just one enzyme but rather a set of enzymes that are acting on the substrate. Regarding co-cultures, this characteristic is further amplified since the crude extract consists of a combination of enzymes produced by the two participating fungi. As such, when evaluating the effect of temperature, what is actually measured is how this physical condition interferes globally with the activity of a specific group of enzymes. Investigating such parameters is important from an industrial standpoint, since commercially available enzyme cocktails are typically composed of crude extracts with the possible addition of exogenous enzymes, rather than purified enzymes alone. Figure 3 depicts the effect of temperature on the enzymatic activity of the co-cultures. Temperatures ranging from 30 to 80 °C were tested to encompass conditions relevant to industrial applications.

Regarding CMCase activity, the monoculture of *T. reesei* RUT-C30 maintained the highest activity between 30 and 50 °C, with an activity peak observed at 50 °C. On the other hand, the CMCase activity of C30 0 h was surpassed by AB + C30 0 h at 60 °C and by AB monoculture at 70 °C and 80 °C. As far as we are aware, this is the first report on the effect of temperature on CMCase activity in *A. brasiliensis*.

Xylanase activity showed the lowest variation according to temperature across all examined cultures. This result was expected, considering that several xylanases which are active at high temperatures have already been reported in the literature, most of which exhibit an optimal activity in the range of 40–60 °C [26]. Concerning co-cultures, AB + C30 0 h produced high xylanase activities between 30 °C and 60 °C which are only comparable to the C30 monoculture.

Regarding pectinase activity, the highest values were obtained by C30 monoculture and AB + C30 0 h, both at 70 °C. *T. reesei* RUT-C30 also presented the highest values for mannanases when assays were conducted at 70 °C. Interestingly, C30 + AB 24 h and C30 + AB 48 h also showed activity comparable to C30 at 70 °C and higher at 80 °C. Overall, mannanase activities obtained in mono- and co-culture formulations were low, indicating that sugarcane bagasse is not, at least under the conditions presented here, an efficient inducer of mannanase activity for the characterized fungi.

The practice of employing a fungus capable of producing β-glucosidase as a partner to supplement *T. reesei* RUT-C30’s deficiency in production of this enzyme is a co-cultivation practice that has been employed with varying degrees of success [18,27,28]. Here, *A. brasiliensis* monoculture showed the highest β-glucosidase activity at a temperature of 50 °C. It is interesting to note that β-glycolytic activity in *A. brasiliensis* remained constant at 50 and 60 °C, with activity in co-culture (AB + C30 0 h) approximately 30% higher at 60 °C than at 50 °C. This fact, along with several other situations presented here, reinforces the conclusion that fungal co-cultures can show unique, complex, and, so far, unpredictable enzymatic characteristics, which are not merely averages between the activities of each of the participating fungi but rather a new composition of the enzymatic cocktail. Interestingly, all cultures where *T. reesei* RUT-C30 was inoculated at intervals of 24 and 48 h prior to *A. brasiliensis* resulted in low β-glucosidase enzyme activities.

β-xylosidase activity was the highlight for *A. brasiliensis* in terms of enzyme secretion. In monoculture, values reached approximately 6 IU/mL at 70 °C, the highest enzyme activity compared to C30 monoculture and all co-culture configurations. In support of our findings, as mentioned earlier, the presence of thermophilic β-xylosidases has also been observed in *A. brasiliensis* grown on agro-industrial residues [29].

For β-galactosidase activity, the highest activities were observed in the co-culture AB + C30 48 h throughout the temperature range. Therefore, a clear synergistic effect of the interaction between both fungi could be observed, with the activity achieved by the co-culture higher than the sum of the activities of the monocultures. With greatest activity at 60 °C, the AB + C30 48 h co-culture showed an activity 46% higher than the sum of the individual activities of *A. brasiliensis* and *T. reesei* RUT-C30 monocultures. The same synergistic effect was also evident, to a lesser extent, at temperatures of 30, 40, and 50 °C. From an industrial point of view, the presence of β-galactosidases that are active at 30 °C or lower temperatures is interesting, as these are typical conditions applied in the dairy industry, where these enzymes are widely employed. The AB + C30 48 h co-culture can be seen as a potential new source of β-galactosidases, enzymes which have enormous economic potential in the food industry [30].

### 3.3. Effect of pH on Enzymatic Activity

**Figure 4:**
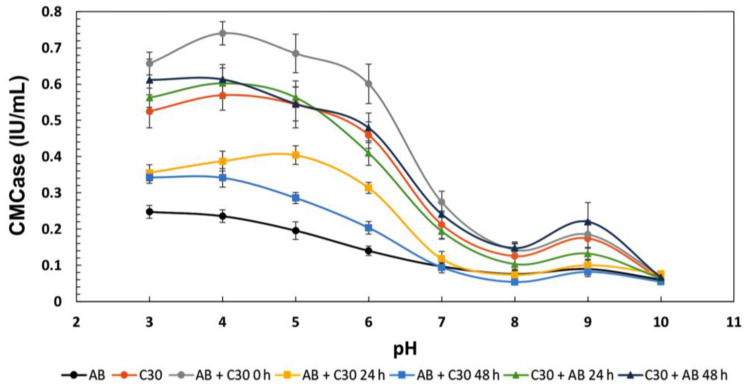
Influence of pH on the CMCase activity of different mono- and co-cultures of *A. brasiliensis* and *T. reesei* RUT-C30, as described in Table 1. The error bars represent the standard deviation between three biological replicates. All experiments showed *p* < 0.05 (ANOVA).

Given its potential importance in co-culture, the effect of pH on the activity of CMCase in the mono- and co-cultures was also investigated, with a pH range between 3 and 10 examined, across one-unit intervals (Figure 4). Once pH conditions were controlled, the simultaneous co-cultivation of *A. brasiliensis* and *T. reesei* RUT-C30 (AB + C30 0 h) showed the highest total values among the cultures. The AB + C30 0 h co-cultivation performed better in the more acidic pH range, as expected and already well documented for enzymes from filamentous fungi. The highest activity of this culture was achieved at pH 4, where a CMCase activity of 0.74 UI/mL was observed. This activity was 32% higher than observed in the monoculture of *T. reesei* RUT-C30, a fungus renowned for its CMCase activity. Throughout the acidic pH range, i.e., pH 3, 4, 5, and 6, the AB + C30 0 h co-culture obtained activities 25%, 32%, 25%, and 33% higher than observed for the monoculture of *T. reesei* RUT-C30, respectively. *T. reesei* RUT-C30 is a strain known for its low production of β-glucosidase, while *A. brasiliensis* has shown to be a potential producer of this enzyme. In this way, the simultaneous cultivation of these two fungi may have created a situation of enzymatic complementarity, resulting in the possible synergy that increased hydrolytic efficiency. Synergy between the various cellulase activities such as endocellulases (CMCases), cellobiohydrolases, and β-glucosidases is crucial for the deconstruction of the cellulose polymer [18]. Therefore, it is possible that the β-glucosidase from *A. brasiliensis* alleviated the product inhibition caused by cellobiose in other cellulolytic enzymes, thus increasing the efficiency of CMC degradation in this experiment.

### 3.4. Saccharification of Sugarcane Bagasse by Crude Extracts of Fungal Co-Cultures

An experiment was conducted to evaluate the ability of crude extracts from mono- and co-cultures to hydrolyze the recalcitrant cell wall of sugarcane bagasse. Saccharification experiments were carried out at 4, 8, 12, and 24 h intervals. In the first 8 h of hydrolysis, all cultures, except for the *A. brasiliensis* monoculture, showed hydrolytic capacity. The co-cultures of *T. reesei* RUT-C30 inoculated 24 and 48 h before *A. brasiliensis* (C30 + AB 24 h and C30 + AB 48 h) exhibited a release of reducing sugars that was 52% (24 h) and 70% (48 h) higher than that observed in the monoculture of *T. reesei* RUT-C30. As mentioned earlier, co-cultures can potentially create a synergistic situation between the enzymatic activities of the participating fungi. Van Dyk and Pletschke [31] analyzed several studies on synergy. They concluded that in more recalcitrant or crystalline substrates (such as the untreated lignocellulosic material employed here), the influence of syner gy is more significant than when compared to substrates more accessible and less resistant to hydrolysis.

Since hydrolysis is performed with crude enzyme extracts, various enzymatic activities are present. In addition to cellulases, other enzymes such as xylanases can also influence the total hydrolysis of lignocellulosic material, acting synergistically with cellulases even though these enzymes act on different polysaccharides [4,17,31]. The degradation of hemicellulosic portions, such as xylan, allows greater access to cellulose by cellulases. It is also possible that co-cultures induce the expression of auxiliary enzymes, which modify the structure of cellulose without hydrolyzing it, thus facilitating access and hydrolysis by other enzymes.

### 3.5. Proteomic Analysis of Mono- and Co-Cultures

As summarized above, specific enzyme assays enabled a comparison of biomass-degrading enzyme activities in *A. brasiliensis* and *T. reesei* RUT-C30 mono- and co-cultures. However, this hypothesis-driven approach certainly misses important global information on the protein composition of the samples. Proteomics, a high-throughput and usually a discovery-driven strategy, can provide a qualitative and quantitative description of the secretomes (i.e., the set of proteins expressed by an organism and secreted into the extracellular space) of the mono- and co-cultures studied here.

Thus, we performed a label-free quantitative bottom-up secretome analysis of the final (9 days) CE of each sample for the samples AB, C30, AB + C30 0 h, AB + C30 24 h and C30 + AB 24 h (see Table 1).

For all samples, including those originating from monocultures of *A. brasiliensis* and *T. reesei* RUT-C30, protein identification searches were based on a concatenated database composed of both *T. reesei* RUT-C30 and *A. brasiliensis* reference proteomes along with common contaminants. As mentioned earlier, this strategy revealed a total of 167 proteins, with 78 from *T. reesei* RUT-C30 and 89 from *A. brasiliensis*. (Supplementary Table S1).

**Figure 5:**
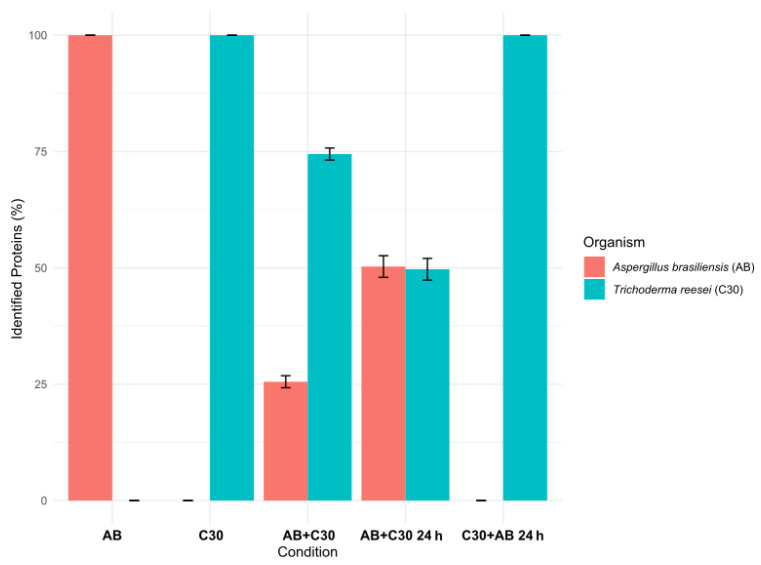
The identification percentage of proteins belonging to either *T. reesei* RUT-C30 or *A. brasiliensis* in mono- and co-cultures. The ratio of identified proteins from each organism was calculated, providing insights into the relative abundance of proteins from each organism under different experimental setups as described in Table 1.

The specificity of the identification strategy was validated by evaluating the list of proteins identified from C30 and AB monocultures. Remarkably, for both monoculture secretomes, all identified proteins (below 1% FDR cutoff) corresponded to UniProt accessions belonging to their specific organism (Figure 5). The absence of cross-species protein identification demonstrates the specificity of the data generated for mono- and co-culture secretomes.

In the AB + C30 0 h co-culture, approximately 75% of the identified proteins belonged to *T. reesei* RUT-C30, while *A. brasiliensis* accounted for around 25% of the identified proteins. This suggests a greater diversity and/or a higher abundance of secreted *T. reesei* RUT-C30 proteins compared to *A. brasiliensis*. By contrast, when *T. reesei* RUT-C30 was inoculated 24 h after *A. brasiliensis* (AB + C30 24 h), the proportion of identified proteins from each organism was approximately 50%, indicating a similar diversity and more comparable abundance of proteins between the two organisms (Figure 5). Interestingly, when *A. brasiliensis* was inoculated 24 h after *T. reesei* RUT-C30 (C30 + AB 24 h), proteins from *A. brasiliensis* were not identified. This could be explained by a higher growth rate of *T. reesei* RUT-C30 compared to *A. brasiliensis*. However, we observed in monocultures that the growth rate of both fungi is similar. It cannot be ruled out that *T. reesei* RUT-C30, which is a genetically improved organism, may display a better adaptation to the competitive conditions of co-cultures.

**Figure 6:**
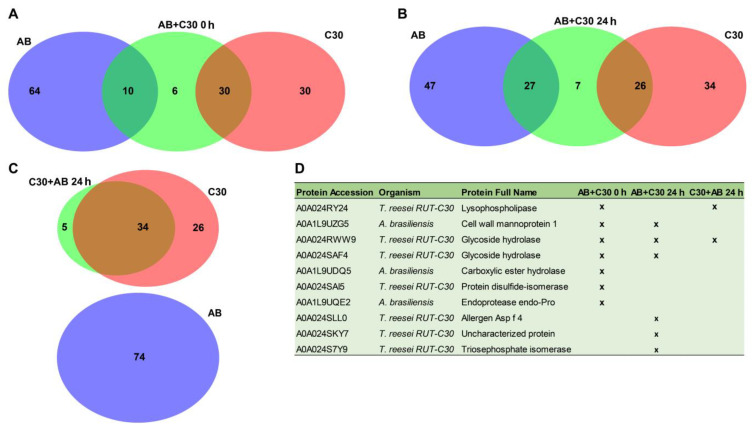
Venn diagram of shared and exclusive proteins identified in the secretomes of different co-culture experimental setups (see Table 1) and monocultures of AB and C30. Panel (**A**) = AB + C30 0 h vs. AB vs. C30; Panel (**B**) = C30 + AB 24 h vs. AB vs. C30; Panel (**C**) = C30 + AB 24 h vs. AB vs. C30. Panel (**D**) = list of proteins identified exclusively in the co-cultures. AB—*A. brasiliensis*; C30 = *T. reesei* RUT-C30.

Differences in protein composition between the co-cultures AB + C30 0 h, AB + C30 24 h and C30 + AB 24 h versus single cultures were represented using Venn diagrams (Figure 6). Additionally, an inset table shows the proteins specifically found in the co-cultures (six for AB + C30 0 h, seven for AB + C30 24 h and five for C30 + AB 24 h). Figure 6C shows that C30 + AB 24 h displays a high number of proteins (34) in common with C30 but with no overlap with AB. This pattern is different to that observed in comparisons with AB + C30 0 h (Figure 6A) and AB + C30 24 h (Figure 6B), which show proteins in common with AB (10 and 27, respectively), although a higher overlap was observed against C30 (30 and 26, respectively).

**Figure 7:**
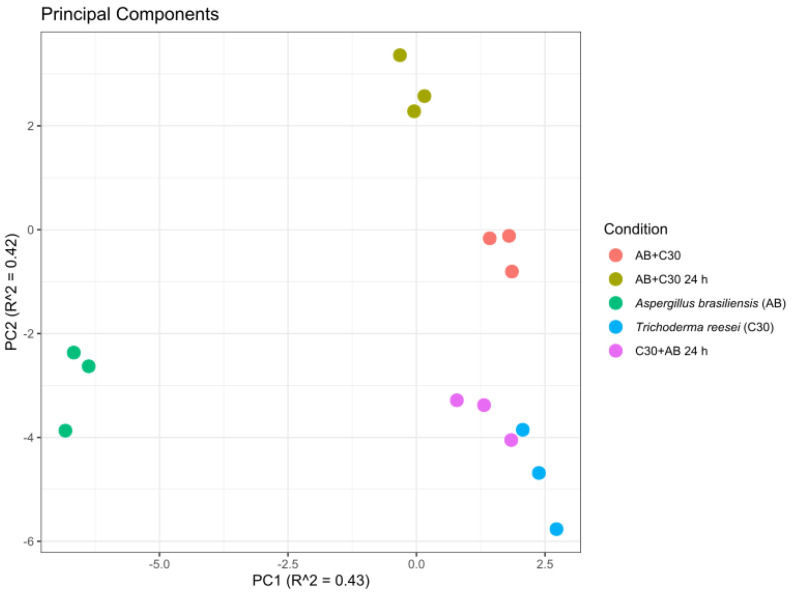
Probabilistic principal component analysis (PPCA) was used to assess similarities among replicates within each group and to identify differences between conditions. Dimension reductions considered both normalized abundance values and number of missing values.

The quantification of the 167 proteins was evaluated by extracted ion chromatograms (XIC) from identified peptides. These were further annotated with match between runs to minimize the number of missing values in each run. This approach relies on the propagation of identification from peptides with similar characteristics in different runs and is highly appreciated for quantitative proteomic comparisons. However, it is important to note that some identification propagation may be misleading, especially in the absence of MS/MS information. To mitigate potential effects, statistical tests were performed only between conditions with similar protein/peptide matrices, namely AB + C30 0 h and AB + C30 24 h. Given the substantial differences in proteins identified when comparing mono- and co-cultures, and particularly in the co-culture condition where *T. reesei* was inoculated first (C30 + AB 24 h), resulting in the identification of only *T. reesei* proteins (Figure 5), these datasets were not submitted to ANOVA tests. Nevertheless, global analysis, such as the probabilistic principal component analysis (PPCA), was employed to assess the similarities among replicates within each group and to identify differences between conditions (Figure 7). PPCA revealed a distinct grouping of all condition replicates, effectively separating single cultures from co-cultures, except for the *T. reesei* RUT-C30 single culture (C30) and C30 + AB 24 h co-culture. The similarity between both samples corroborates the substantial overlap in the identified proteins between the C30 + AB 24 h co-culture and C30 monoculture (Figure 6B). In addition, it was shown that all proteins identified in C30 + AB 24 h belonged to *T. reesei* RUT-C30 (Figure 5). Overall, the PPCA demonstrated differences between conditions and suggested similarities between C30 and C30 + AB 24 h conditions.

Quantitative comparisons were conducted to assess the differences between different time points of *T. reesei* RUT-C30 inoculum in the co-culture (AB + C30 0 h vs. AB + C30 24 h). The results of this test revealed 58 regulated proteins (ANOVA, *p*-value < 0.05), with 23 proteins up-regulated in AB + C30 0 h and 35 proteins up-regulated in AB + C30 24 h (Supplementary Table S2). The proteins that showed significantly higher abundance in the AB + C30 0 h condition belonged exclusively to *T. reesei* RUT-C30. In contrast, the up-regulated proteins in AB + C30 24 h originated from *A. brasiliensis*. These findings are consistent with the identification evaluations, which suggested a higher ratio of *T. reesei* RUT-C30-identified proteins in the co-culture with simultaneous inoculation (AB + C30 0 h), as shown in Figure 5.

Collectively, these data indicate that, not only in terms of diversity but also in terms of abundance, *T. reesei* RUT-C30 proteins prevail over *A. brasiliensis* when the inoculum occurs simultaneously. On the other hand, when *T. reesei* RUT-C30 inoculum is introduced 24 h later, *A. brasiliensis* may contribute in a greater proportion to the co-culture, both in terms of diversity and abundance of its secreted proteins. Furthermore, analysis of interactions and functional characterization of the regulated proteins revealed that they were significantly involved in polysaccharide metabolic and catabolic processes (see Supplementary Table S3). This is evident from the interactions formed among these regulated proteins, including glucanases, mannanases, and beta-glucosidases from *A. brasiliensis* in the AB + C30 24 h condition, and alpha-galactosidases, xyloglucanases, endopolygalacturonases, among others, from *T. reesei* RUT-C30 in the AB + C30 0 h secretome. Additionally, two regulated proteins in *A. brasiliensis* were found to be related to serine proteases (see Supplementary Table S3). These classifications suggest that *T. reesei* RUT-C30 may contribute less to the polysaccharide metabolic processes when this fungus is inoculated 24 h after *A. brasiliensis*. However, this decrease in the contribution of *T. reesei* RUT-C30 might be compensated for and even enhanced by polysaccharide metabolic enzymes from *A. brasiliensis*.

## 4. Conclusions

The modern world is grappling with a significant increase in environmental problems, largely stemming from a linear fossil-based economy. Consequently, there is an urgent need for transition to a circular bioeconomy, emphasizing the utilization of renewable natural resources and waste minimization. In this context, plant biomass represents a promising alternative to fossil fuels, with lignocellulolytic enzymes essential for conversion of this biomass into fermentable sugars [32]. This study provides a comprehensive characterization of the proteins secreted by the cellulase production workhorse, *T. reesei* RUT-C30, together with *A. brasiliensis*, a fungus directly isolated from soil samples in the Brazilian Cerrado. A crucial aspect of the research involves investigating the co-cultures of both fungi under various conditions, such as co-inoculation at 0 h and an alternative order of inoculation in the culture media. This approach yields diverse protein cocktails, extensively examined for biomass-degrading enzyme activities and a label-free quantitative proteome analysis. The co-cultivation of fungi presents a promising future for enzyme production through the natural synergies between different fungal species [32,33]. This approach can lead to the development of more efficient, cost-effective and sustainable enzyme production processes, including enhanced enzyme yield and diversity. It is important to highlight that fungi can engage in synergistic interactions where the presence of one species enhances the enzyme production of another. This can occur through various mechanisms, including the exchange of signaling molecules, sharing of metabolic by-products, or direct physical interactions. These synergies can significantly boost overall enzyme production [32].

The results reveal distinctive enzymatic and proteomic profiles for each co-culture, underscoring the significance of considering this parameter in the design of co-culture experiments. Notably, this work contributes considerably to the understanding of *A. brasiliensis*, which has been inadequately studied thus far. The findings represent a substantial contribution to the field of fungal co-cultures by addressing characterization parameters lacking in international literature. However, it is crucial to acknowledge that the cultivation conditions established in this study are suboptimal for enzymatic production. The crude and highly recalcitrant nature of the employed substrate hinders hydrolysis and the subsequent release of sugars to the fungi. From a future perspective, the utilization of pre-treated substrates and optimization of cultivation conditions is relevant. Furthermore, this study enables an assessment of the contribution of each fungal species during co-culture, determining the number of identified proteins from each organism. The investigation also provides an approach for the specific identification of proteins originating from each of the different microorganisms present in a co-culture.

## Figures and Tables

**Table 1:** Configurations of monocultures and co-cultures employed in this study.

Experimental Setup (Abbreviation)	Intervals between Inoculations
*A. brasiliensis* (AB) *	-
*T. reesei* RUT-C30 (C30) *	-
*A. brasiliensis* + *T. reesei* RUT-C30 (AB + C30 0 h) *	-
*A. brasiliensis* + *T. reesei* RUT-C30 (AB + C30 24 h) *	24 h
*A. brasiliensis* + *T. reesei* RUT-C30 (AB + C30 48 h)	48 h
*T. reesei* RUT-C30 + *A. brasiliensis* (C30 + AB 24 h) *	24 h
*T. reesei* RUT-C30 + *A. brasiliensis* (C30 + AB 48 h)	48 h

The abbreviation of each experiment reflects inoculation order, with the first one (AB or C30) representing the first strain to be inoculated, and the second one the coculture strain introduced after a specified interval in hour (h). For enzyme activity assays, aliquots were collected from each sample after periods of 1, 3, 5, 7, and 9 days. For proteomics analysis, only the final CEs (9 days) were analyzed, for the samples marked with an asterisk (*).

## Data Availability

The original contributions presented in the study are included in the article/Supplementary Material, further inquiries can be directed to the corresponding author.

## Supplementary Materials

The following supporting information can be downloaded at: https://www.mdpi.com/article/10.3390/jof10100677/s1, Table S1: Identified proteins from the secretomes of *Aspergillus brasiliensis* and *Trichoderma reesei* RUT-C30 mono- and co-cultures; Table S2: ANOVA tested and regulated proteins between different times of *Trichoderma reesei* RUT-C30 inoculum (0 h and 24 h after *Aspergillus brasiliensis*); Table S2.1: Proteins up-regulated in condition AB + C30 0 h; Table S2.2: Proteins up-regulated in condition AB + C30 24 h; Table S3: String overrepresentation analysis of regulated proteins between different times of *Trichoderma reesei* RUT-C30 inoculum in the co-culture. The proteins considered as regulated was those that presented ANOVA *p*-value < 0.05 between conditions AB + TR and AB + TR (24 h).
